# Glucose-Lowering Drugs with Proven Cardiovascular Benefit Following Acute Coronary Syndrome in Patients with Type 2 Diabetes: Treatment Gaps and Outcomes

**DOI:** 10.3390/jcm13185541

**Published:** 2024-09-19

**Authors:** Ibrahim Naoum, Walid Saliba, Ofra Barnett-Griness, Amir Aker, Barak Zafrir

**Affiliations:** 1 Department of Cardiology, Lady Davis Carmel Medical Center, Haifa 3436212, Israel; naoum.i.md@gmail.com (I.N.); akeramir90@gmail.com (A.A.); 2 Community Medicine and Epidemiology, Lady Davis Carmel Medical Center, Haifa 3436212, Israel; 3 Faculty of Medicine, Technion—Israel Institute of Technology, Haifa 3525433, Israel; 4 Statistical Unit, Lady Davis Carmel Medical Center, Haifa 3436212, Israel

**Keywords:** type 2 diabetes mellitus, SGLT2 inhibitors, GLP1 receptor agonists, acute coronary syndrome, real world, drug prescriptions

## Abstract

**Background:** Real-world data on the implementation and prognostic impact of glucose-lowering drugs with proven cardiovascular benefits in patients with type 2 diabetes (T2D) following acute coronary syndrome (ACS) are limited. We investigated the utilization and treatment patterns of sodium–glucose contrasporter-2 inhibitors (SGLT2Is) and glucagon-like peptide-1 recepto-agonists (GLP1RAs) in patients with T2D experiencing ACS and analyzed their association with mortality and major adverse cardiovascular events (MACEs) including recurrent ACS, acute revascularization, heart failure, or ischemic stroke. **Methods**: We carried out a retrospective analysis of 9756 patients with T2D from a nationwide healthcare organization in Israel who were hospitalized with ACS between 01/2019 and 01/2022. Drug prescriptions were estimated pre-hospitalization, 90 days, and 1 year following hospitalization. The association between SGLT2I and/or GLP1RA treatment with MACE and mortality was investigated using a time-dependent Cox regression analysis with multivariable adjustment. **Results**: The prescription rates (pre-hospitalization, 90 days, and 1 year post-hospitalization) of GLP1RAs were 13%, 13.2%, and 18%, and those of SGLT2Is were 23.9%, 33.6%, and 42.7%, respectively. At 1 year, 13.9% of patients were prescribed both treatments. The use of SGLT2Is and/or GLP1RAs was higher in younger age groups and increased from 2019 to 2021 (38.1% to 59.2%). The adjusted hazard ratio for the association of pre- or post-hospitalization SGLT2I and/or GLP1RA treatment with mortality and MACE was 0.724 (0.654–0.801) and 0.974 (0.909–1.043), respectively. **Conclusions**: In the real-world practice of treating patients with T2D experiencing ACS, the implementation of SGLT2Is, particularly GLP1RAs, was suboptimal when prescribed both early and 1 year following hospitalization, emphasizing the need to improve medical care. Treatment with SGLT2Is and/or GLP1RAs was associated with a favorable impact on mortality but not MACE.

## 1. Introduction

The prevalence of type 2 diabetes mellitus (T2D) is consistently and globally increasing [1]. Diabetes is an important risk factor for cardiovascular disease, which remains the leading cause of morbidity and mortality in patients with diabetes [2]. Coronary artery disease (CAD) in patients with T2D is often more complex and diffused, initiates at a younger age, and progresses faster [3]. Diabetes confers an excess risk following acute coronary syndromes (ACSs) and is associated with an increase in post-procedural complications, the need for recurrent interventional procedures, heart failure, and mortality [4,5]. In recent years, new prescriptions for diabetes were introduced, with proven efficacy in improving cardiovascular outcomes in patients with or at a high risk of developing CAD [6,7]. These include sodium–glucose cotransporter-2 inhibitors (SGLT2Is), which were shown to reduce heart failure hospitalizations and mortality as well as to preserve kidney function [8,9,10], and glucagon-like peptide-1 receptor agonists (GLP1RAs) to which anti-atherogenic effects are attributed [11,12,13]. Both treatments are recommended in recent guidelines and consensus documents in patients with T2D and atherosclerotic cardiovascular disease (ASCVD) to reduce cardiovascular events independent of the glycosylated hemoglobin level or concomitant glucose-lowering medications [2,6,7].

Despite the acknowledged evidence-based clinical benefits of both GLP1RAs and SGLT2Is in patients with T2D, real-world data on the implementation of both treatments following hospitalization with ACS are limited.

Unlike randomized clinical trials, numerous patient, provider, and health system barriers may influence the initiation and adherence to medications in routine clinical practice and hinder the penetrance of novel drugs [14]. A recent study found that empagliflozin initiated early after acute myocardial infarction reduced the risk of heart failure in patients with left ventricular dysfunction or congestion, though it did not lead to a significant reduction in the primary composite outcome [15,16]. It should be noted however, that a lack of prescription of disease-modifying drugs at discharge from hospitalization may delay treatment and adversely impact clinical outcomes, as it is less likely that an evidence-based medication will be initiated at the discretion of the primary care physician in the community [17].

In light of the above considerations, the aim of the present study was to investigate the implementation of glucose-lowering drugs with proven cardiovascular benefits in clinical practice for the treatment of patients with T2D experiencing ACS. We analyzed the prescription rates and treatment patterns of SGLT2Is and GLP1RAs at admission and following hospitalization with ACS. Furthermore, we aimed to examine the association between the use of SGLT2Is and/or GLP1RAs and adverse cardiovascular events and mortality following ACS in patients with T2D.

## 2. Materials and Methods

### 2.1. Data Source and Study Population

This study is based on data from the computerized database of Clalit Health Services (CHS), which provides inclusive healthcare for more than half of the Israeli population (~4.7 million members). All patients were members of CHS, and we had full access to patients’ computerized data. The electronic medical record database of CHS includes data from multiple sources: records of primary care physicians, community specialty clinics, hospitalizations, laboratories, and pharmacies. A registry of chronic disease diagnoses is compiled from these data sources. Diagnoses are captured in the registry by diagnosis-specific algorithms using ICD-9 codes, text reading, laboratory test results, and disease-specific drug usage.

**Figure 1:**
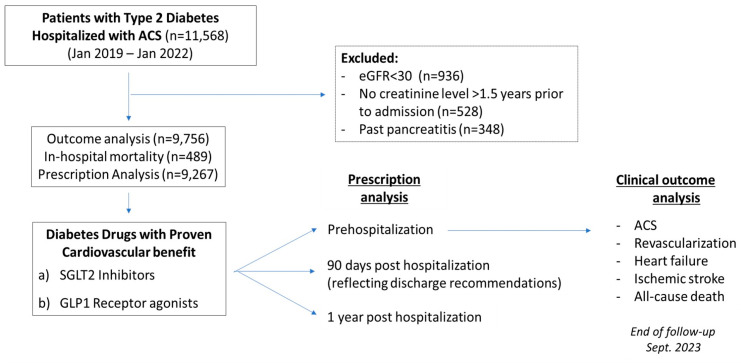
Study population outline.

The study cohort consisted of all adult CHS members >18 years with a pre-existing diagnosis of T2D who were hospitalized with an ACS event between the period of January 2019 and January 2022. Patients with type 1 diabetes, a reduced estimated glomerular filtration rate (eGFR) <30 mL/min/1.73 m^2^, and those with prior events of pancreatitis were excluded. Study participants were followed-up with until reaching study outcomes, death, or until the end of follow-up at 30 September 2023, whichever came first. For the analysis of drug prescription following hospitalization, a cohort of patients was defined including only patients who had been discharged alive from the index hospitalization, and follow-up commenced from the discharge date, whereas the whole study cohort was used for the analysis of the clinical outcomes as defined below. This study was approved by the CHS Ethics Committee in accordance with the Declaration of Helsinki, and the need for individual patient consent was waived due to the retrospective design of this study. This study’s outline is presented in Figure 1.

### 2.2. Study Variables and Definitions of Terms

The following baseline data were retrieved for each patient from the computerized database of CHS: demographic and descriptive variables, socioeconomic status (SES) defined based on the SES score of the clinic neighborhood as determined by the Israel Central Bureau of Statistics, presence of cardiovascular risk factors and comorbidities, selected chronic medical conditions, laboratory values, and medication use. In addition, ACS type (defined as unstable angina or myocardial infarction) and prior diagnosis of CAD and heart failure were documented. The diagnosis of diabetes was retrieved from the CHS chronic disease registry that relies on different sources, including a clinical diagnosis of diabetes (ICD-9 code 250), HbA1c ≥ 6.5%, and diabetes-specific drug usage (ATC code A10). Data on medication use are believed to be complete because of the very low copayment required in CHS, making it unlikely that prescription medications would be purchased in non-CHS pharmacies. Diabetes drugs with positive cardiovascular outcome trials included SGLT2Is (empagliflozin and dapagliflozin) and GLP1RAs (liraglutide, dulaglutide, and semaglutide) (6). The use of metformin and insulin was also documented. Pre-hospitalization medication prescriptions were defined as drugs prescribed most adjacent and up to 1 year prior to the index hospitalization.

Study outcomes were defined as the cumulative incidence of SGLT2I and/or GLP1RA prescription at 3 months post-hospitalization, reflecting discharge recommendations, and 1 year following the ACS event, reflecting additional treatment initiation at the community by the primary care or consulting physicians. In addition, all-cause mortality and major adverse clinical events (MACEs) during follow-up were analyzed in relation to utilization of SGLT2Is and/or GLP1RAs pre- and post-hospitalization with ACS. MACE was defined as a composite event including recurrent hospitalization for ACS, acute revascularization, heart failure, or ischemic stroke. Data on adverse cardiovascular events were retrieved from the CHS hospitalizations database and were defined according to the specific ICD-9 codes of primary discharge diagnoses. Data on vital status were retrieved from the Ministry of Interior.

### 2.3. Data Analysis

Statistical analyses were performed using version 9.4 SAS software (SAS Institute, Cary NC). For all analyses, *p* < 0.05 for the two-tailed tests was considered statistically significant. Continuous data are reported as median values and interquartile range (IQR), and categorical variables are presented as numbers and percentages. Comparisons between two categorical variables were performed using the chi-square test (or Fisher’s exact test, as appropriate), and continuous variables were compared using Wilcoxon test. For drug prescription analysis, we included all patients surviving hospitalization (*n* = 9267). Drug prescriptions over 90 days and 1 year following hospitalization were estimated and graphically presented using a cumulative incidence function analysis, with death prior to drug prescription serving as competing risk. The difference in the use of glucose-modifying drugs before versus after hospitalization with ACS was tested employing the bootstrap method. Additional analyses were performed to evaluate the cumulative incidence of drug prescriptions over time according to (1) age groups, (2) the year of hospitalization (2019 vs. 2020 vs. 2021), (3) pre-hospitalization use of SGLT2Is and/or GLP1RAs, and (4) history of CAD or heart failure prior to hospitalization. Comparison between curves was performed using the Gray’s test. Furthermore, shift analysis of drug prescriptions from pre-hospitalization users to any time during first year post-hospitalization was performed.

For clinical outcome analysis (MACE or mortality), we included all ACS patients admitted to the hospital (*n* = 9756). Cox proportional hazard regression models were used to assess the association between prescriptions of SGLT2I and/or GLP1RA therapeutic groups at pre- or post-hospitalization, analyzed as time-dependent exposure and time to MACE or death during follow-up (each outcome was reported separately and also as a composite MACE/death outcome), and crude and adjusted hazard ratios (HRs) and 95% confidence intervals (CI) were estimated, with the group of patients not receiving SGLT2Is and/or GLP1RAs serving as the reference category. Variables for the multivariable adjustment included age, sex, ethnicity, country district, socioeconomic status, obesity, hypertension, hyperlipidemia, active smoking, hemoglobin level, total cholesterol, high-density and low-density lipoprotein cholesterol, triglycerides, eGFR, diabetes duration (years), body mass index, insulin therapy, diabetic microvascular complications (neuropathy, nephropathy, or retinopathy), chronic obstructive pulmonary disease, chronic kidney disease, prior stroke, peripheral artery disease, active malignancy, ischemic heart disease, and chronic heart failure.

## 3. Results

### 3.1. Study Population and Patients’ Characteristics

A total of 11,568 adult patients with T2D insured by CHS were hospitalized with ACS during the study period. We excluded patients with reduced kidney function (eGFR < 30 mL/min/m^2^), those lacking documentation of creatinine levels, and patients with a history of pancreatitis. The remaining 9756 patients constituted the overall study cohort (Figure 1). The median age of the patients was 70 years (IQR 61.8–79 years), and 30.6% were females. ACS was classified as unstable angina in 28.4% and acute myocardial infarction in 71.6%. The median duration of diabetes was 14 years (IQR, 7–19 years), with 30% of the patients being treated by insulin therapy. Concomitant cardiovascular risk factors included hypertension and hyperlipidemia, which were observed in most subjects, as well as obesity and active smoking, which were observed in about half of the patients. The baseline demographic and clinical characteristics of the study population are shown in Table 1. In addition to CAD, stroke and peripheral artery disease were each evident in about one-fifth of the study patients, and a quarter had a baseline diagnosis of heart failure.

### 3.2. Utilization of Glucose-Modifying Drugs with Cardiovascular Benefit

A drug prescription analysis was performed on the discharged sub-cohort, consisting of 9267 patients. GLP1RAs were used by 13% of the patients with T2D prior to admission. The cumulative incidence rate of prescriptions was similar at 3 months post-discharge (13.2%) and increased to 18.8% 1 year post-hospitalization (*p* < 0.001) (Table 2). SGLT2Is were prescribed to 23.9% of the patients prior to hospitalization, with the cumulative incidence rate of utilization already higher than the baseline at 3 months (33.6%) and further increasing 1 year (42.7%) post-discharge (*p* < 0.001 for both comparisons). Only a minority of the study population was treated by both SGLT2Is and GLP1RAs, with combined drug prescriptions increasing from 7.3% at baseline to a cumulative incidence rate of 13.9% 1 year following hospitalization.

**Figure 2:**
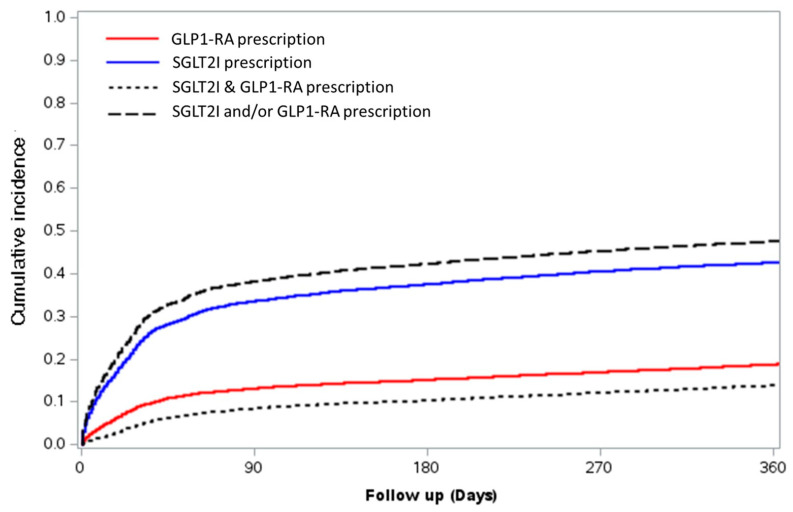
Cumulative 1-year incidence of SGLT2I and/or GLP1-RA prescriptions following hospitalization with ACS.

Overall, 1 year following discharge from ACS hospitalization, the estimated cumulative incidence rate for patients with T2D treated by any of the two therapeutic groups (SGLT2Is and/or GLP1RAs) was 47.6%. The cumulative incidence of GLP1RA and/or SGLT2I prescriptions 1 year following ACS hospitalization is graphically presented in Figure 2.

**Figure 3:**
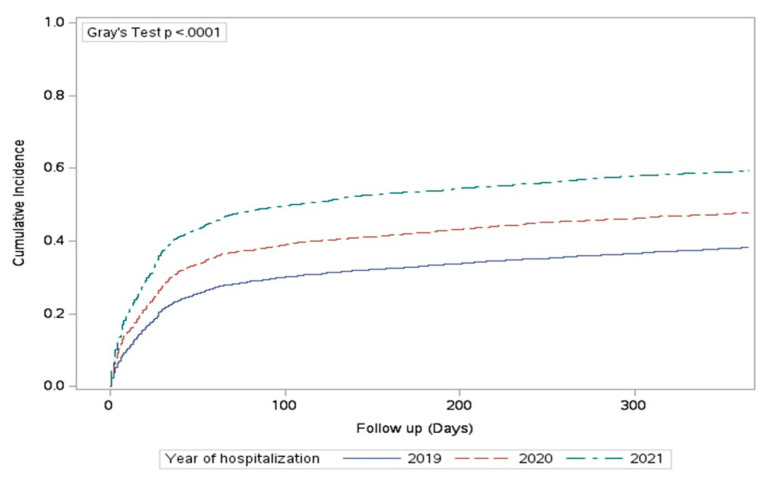
The cumulative 1-year incidence of GLP1-RA and/or SGLT2I prescriptions according to the year of hospitalization.

Most of the patients who were treated at baseline by any of the SGLT2 and/or GLP1RA drugs retained their drug prescriptions 1 year following acute hospitalization (92.7%). On the other hand, of those without prior use of these prescriptions, only 28.6% initiated drug therapy during the 1-year follow-up (Supplementary Materials, Figure S1). The cumulative incidence of GLP1RA and/or SGLT2I drug prescriptions following ACS was similar in those with or without a baseline diagnosis of CAD or heart failure (47.1% vs. 48.7%; *p* = 0.10). In addition, the cumulative incidence rate of prescriptions was significantly higher with the decrease in the patients’ age (Supplementary Materials, Figure S2). Furthermore, a significant increase in the 1-year cumulative incidence rate of SGLT2 and/or GLP1RA prescription rates was observed with the progression of the year of hospitalization: it was 38.1%, 47.7%, and 59.2% during the years 2019, 2020, and 2021, respectively (*p* < 0.001; Figure 3).

A shift analysis comparing drug prescriptions before and after hospitalization is presented in the Supplementary Materials in Table S1. More patients treated prior to hospitalization with only GLP1RA than those treated with only SGLT2Is added the second therapeutic group 1 year following hospitalization (37.4% versus 17.1%). In addition, of those treated at admission with both GLP1RAs and SGLT2Is, 14.6% patients stopped one of the prescriptions during the follow-up period after discharge.

### 3.3. Adverse Cardiovascular Outcomes and Mortality

During a median (IQR) follow-up period of 38.5 (28.5–47.8) months, 2385 patients (24.4%) died and 4648 (47.6%) experienced MACEs. After a multivariable adjustment, the HR (95% CI) for mortality associated with pre- or post-hospitalization treatment with SGLT2Is and/or GLP1RAs was 0.724 (0.654–0.801), with *p* < 0.0001, and for the outcome of MACEs, the cause-specific adjusted HR was 0.974 (0.909–1.043), with *p* = 0.4511, compared to those not treated by any of the two therapeutic groups. The adjusted HR for a combined endpoint of MACE or mortality associated with any pre- or post-hospitalization use was 0.884 (0.831–0.940), with *p* < 0.001 (Table 3).

Variables for the multivariable adjustment included age, sex, ethnicity, country district, socioeconomic status, obesity, hypertension, hyperlipidemia, active smoking, hemoglobin level, total cholesterol, high-density and low-density lipoprotein cholesterol, triglycerides, eGFR, diabetes duration (years), insulin therapy, diabetic microvascular complications (neuropathy, nephropathy, or retinopathy), chronic obstructive pulmonary disease, chronic kidney disease, prior stroke, peripheral artery disease, active malignancy, ischemic heart disease and chronic heart failure.

## 4. Discussion

In a nationwide cohort of individuals with T2D admitted for ACS during a 3-year period in Israel, our study revealed a suboptimal utilization of SGLT2Is and/or GLP1RAs during both 3 months and 1 year following hospitalization. More than half of the patients lacked a prescription for either drug category even up to 1 year following hospitalization with ACS. While a progressive rise in the use of either SGLT2Is or GLP1RAs was observed during the study period, the overall usage remained limited, particularly among the elderly. Remarkably, even five years after the disclosure of the first cardiovascular outcome trials demonstrating the cardiovascular benefits of SGLT2Is and GLP1RAs [8,9,10,11,12,13], a substantial portion of patients with T2D following ACS—over 40%—did not receive a glucose-lowering drug with proven cardiovascular benefit. Furthermore, therapy with SGLT2Is and/or GLP1RAs pre- or post-hospitalization with an ACS event was associated with a favorable impact on mortality but not MACEs (Graphical Abstract). The persistence of underutilization of drug therapy occurred despite continuous updates and recommendations from major medical societies [18,19,20].

Our findings align with recent data on the implementation of SGLT2Is and GLP1RAs among patients with T2D with established ASCVD [21,22,23,24] and expand the results to a population with T2D experiencing ACS. In a multicenter, retrospective real-world cohort study involving 88 healthcare systems in the United States (US), the utilization of either SGLT2Is or GLP1RAs increased from 11.4% in 2018 to only 23.3% in 2021 [22]. This study revealed that many individuals with ASCVD and T2D receiving medication therapy were using drugs without proven cardiovascular benefits. Hofer et al. conducted a study assessing real-world data on the clinical implementation of SGLT2Is and GLP1RAs among patients with T2D admitted to a cardiology ward in Vienna with established CAD, ACS, or congestive heart failure between 2014 and 2020 [22]. Despite a steady increase in prescriptions over the 6-year study period, only 27.8% of patients were prescribed SGLT2Is and 7.6% were prescribed GLP1RAs in 2019. Similarly, Nair et al. demonstrated a progressive rise in the use of SGLT2Is and GLP1RAs from 2015 to 2019 among patients with T2D and with or at risk for cardiovascular disease in the US [23]. However, the overall utilization of these agents remained low, especially among those aged 65 years and above.

Despite the utilization rate nearly doubling after one year, less than 15% of the patients received prescriptions for combination therapy with SGLT2Is and GLP1RAs. The latest guidelines strongly recommend (Class I, Level A) the use of either SGLT2Is or GLP1RAs to mitigate cardiovascular events in T2D and ASCVD independent of glycemic control and concomitant glucose-lowering medication [7]. However, the guidelines do not relate to or mandate the simultaneous use of both drug categories. In a recent prospective observational study led by Marfella et al. [25], individuals with T2D admitted for ACS and treated with a combination of SGLT2Is and GLP1RAs exhibited a reduced incidence of a composite outcome comprising ACS, hospitalization for heart failure, and all-cause mortality compared to those receiving SGLT2Is or GLP1RAs alone. This synergistic positive impact persisted independently of glycemic control and appeared to be conferred by the dual influence of SGLT2Is on reducing heart failure hospitalization and GLP1RAs on mitigating ACS. Despite the promising and encouraging data, challenges in optimizing combination therapy may persist, influenced in part by insufficient patient education, physician awareness of updated guidelines, and the financial barriers associated with the high cost and limited reimbursement for these drugs.

Our study offers valuable insights into the implementation patterns of diabetic drugs with proven cardiovascular benefits. Notably, over 90% of patients who were initially treated with either drug category (SGLT2Is and/or GLP1RAs) before an ACS event have maintained their prescriptions 1-year post-discharge. However, fewer than 30% of patients without prior exposure initiated drug therapy during the one-year follow-up, suggesting that potential prescribers in both primary and specialty care may not be fully aware of the cardioprotective benefits of these drugs. This is further emphasized by the similarity in the cumulative incidence of GLP1RA and/or SGLT2I drug prescriptions following ACS among individuals with or without a history of CAD or heart failure diagnosis. In a multinational cross-sectional study, Mosenzon et al. showed similar utilization patterns for SGLT2Is and/or GLP1RAs among patients with or without cardiovascular disease [26]. These observed treatment patterns may be further attributed to clinical inertia and the reluctance of physicians to prescribe or recommend the early initiation of SGLT2Is or GLP1RAs following ACS due to concerns about potential adverse effects and, possibly, a lack of awareness regarding their beneficial effects shortly after ACS [27]. In the EMMY trial, the initiation of SGLT2Is shortly after an acute myocardial infarction significantly reduced the N-terminal pro-hormone of brain natriuretic peptide (NT-proBNP) and had positive effects on structural and functional echocardiographic parameters after 26 weeks of treatment without notable safety concerns compared to the placebo [28]. A secondary analysis of the EMMY trial demonstrated that the very early administration of SGLT2Is following acute myocardial infarction does not exhibit adverse safety signals and appears equally effective in reducing NT-proBNP and improving functional and structural left ventricular markers [29].

Similar to our research, the CAPTURE study found an increased burden of cardiovascular disease in patients with T2D (34.8%) across 13 countries, with the highest rate in Israel at 56.5% [26]. Most of these patients were not treated with SGLT2Is nor GLP1RAs. Our study demonstrates a notable reduction in the combined adverse outcome of MACEs or mortality linked to the administration of SGLT2Is and/or GLP1RAs pre- or post-hospitalization. This risk reduction, primarily driven by a significant decrease in mortality, highlights the potential benefits of these medications in improving patient outcomes. In a recent retrospective study on patients with acute myocardial infarction, pre-hospitalization chronic treatment with either SGLT2Is or GLP1RAs correlated with a reduced composite outcome, including in-hospital mortality, acute heart failure, and acute kidney injury requiring renal replacement [30]. Several mechanisms have been proposed to account for the positive impact exerted by GLP1RAs and SGLT2Is on clinical outcomes post myocardial infarction. GLP1Ras have proven effective in enhancing myocardial function post acute myocardial infarction and has demonstrated the ability to reduce the infarct size in both clinical and preclinical settings [31,32,33]. In an experimental model, the administration of liraglutide prior to inducing myocardial infarction led to a reduction in infarct size and an improvement in survival [31]. This effect may be attributed to the activation of pro-survival pathways in cardiomyocytes. Moreover, prolonged treatment with GLP1RA demonstrated the capacity to decrease blood pressure and levels of atherogenic lipoproteins [34,35]. This may potentially result in a favorable hemodynamic profile and a diminished burden of coronary atherosclerosis during the acute myocardial infarction event. Likewise, the use of SGLT2Is has been observed to protect cardiac contractile function in the context of myocardial ischemia–reperfusion injury [36]. In addition, a large body of experimental evidence suggests that SGLT2Is exert vasculoprotective effects involving mechanisms that may act independently of their glucose-lowering properties. These include, among others, NO-mediated attenuation of oxidative stress, the preservation of mitochondrial function, the alleviation of microvascular dysfunction, the inhibition of inflammation, and the restoration of energy homeostasis [37].

The noted reduction in mortality risk linked to the pre- and post-hospitalization use of SGLT2Is and/or GLP1RAs underscores the potential cardiovascular advantages of initiating these medications early. Our findings emphasize the need for increased awareness, education, and proactive measures among healthcare providers to optimize the utilization of SGLT2Is and GLP1RAs in individuals with T2D experiencing ACS, ensuring that patients receive the full spectrum of cardiovascular benefits these medications may offer. The observation of a positive impact on overall mortality but not on MACEs in our study raises intriguing questions regarding the underlying mechanisms. SGLT2I and GLP1RA drugs may exert their effects through pathways that are more directly related to mortality, such as reducing inflammation and improving endothelial function, rather than mechanisms that impact non-fatal events such as recurrent angina and revascularization. Furthermore, the time course of the effects of these drugs and the heterogeneity of the study population in terms of the baseline risk and treatment response are also factors that could contribute to the observed discrepancy. The effects of GLP1RAs and SGLT2is on mortality and MACE outcomes may have different time courses, with a more significant impact on mortality early on following the acute coronary event, but a more delayed or gradual impact on reducing MACEs. Further research is needed to elucidate the specific mechanisms involved and their impact on cardiovascular outcomes.

The current study presents several important limitations that warrant consideration. First, the observational retrospective design inherently introduces the possibility of selection bias and the influence of unmeasured residual confounding variables. In addition, the causality between drug therapy and clinical outcomes cannot be determined. Second, the prescription patterns of GLP1RAs and SGLT2Is may have been influenced by insurance criteria, potentially introducing a selection bias that could identify a population at a higher risk. Third, the substantial increase in the clinical use of semaglutide in the past 2 years, driven by positive outcome studies, may not be fully reflected in our study. Fourth, our dataset lacked information on important clinical variables in patients with ACS such as left ventricular ejection fraction and data on the type of revascularization. We also lacked data on the attainment of optimal treatment targets of modifiable risk factors following ACS, which is essential for improving clinical outcomes. Finally, it is possible that the COVID-19 pandemic may have contributed to the therapeutic inertia, with a lack of initiation or intensification of guideline-directed glucose-lowering drugs with proven cardiovascular benefit in the early phase of the study. These limitations underscore the need for a cautious interpretation of our findings and the acknowledgment that certain variables were not within the scope of our analysis.

## 5. Conclusions

In Israel’s real-world clinical practice, there was a suboptimal implementation of guideline-recommended therapies with SGLT2Is, particularly GLP1Ras, in patients with T2D sustaining ACS both early and one year following hospitalization. Therapy with SGLT2Is and/or GLP1RAs pre- or post-hospitalization with ACS was associated with a favorable impact on mortality but not MACEs. These findings emphasize the need to improve medical care in patients with T2D following ACS.

## Figures and Tables

**Table 1:** Baseline clinical characteristics.

		Baseline Treatment		
Variable	Overall (*n* = 9756)	None (*n* = 6929)	SGLT2Is and/or GLP1RAs (*n* = 2827)	*p* Value
Age (years)	70 (61.8, 79)	71.9 (62.8, 81.2)	67 (60.3, 73.3)	<0.001
Sex (Female)	2984 (30.6%)	2237 (32.3%)	747 (26.4%)	<0.001
Ethnicity: Arab	2584 (26.5%)	1759 ((25.4%)	825 (29.2%)	<0.001
Socioeconomic status:				0.802
- Low	4259 (44%)	3033 (44.1%)	1226 (43.6%)	
- Middle	3870 (40%)	2744 (39.9%)	1126 (40%)	
- High	1557 (16.1%)	1095 (15.9%)	462 (16.4%)	
Body mass index, kg/m^2^ (*n* = 9751)	28.7 (25.6, 32)	28.3 (25.3, 31.6)	29.6 (26.7, 32.9)	<0.001
Obesity	5414 (55.5%)	3511 (50.7%)	1903 (67.3%)	<0.001
Hypertension	8322 (85.3%)	5822 (84%)	2500 (88.4%)	<0.001
Hyperlipidemia	9419 (96.5%)	6628 (95.7%)	2791 (98.7%)	<0.001
Active smoking	4789 (49.1%)	3323 (48%)	1466 (51.9%)	<0.001
COPD	1370 (14.0%)	1020 (14.7%)	350 (12.4%)	0.003
Cerebrovascular accident	1892 (19.4%)	1377 (19.9%)	515 (18.2%)	0.061
Peripheral artery disease	2107 (21.6%)	1500 (21.6%)	607 (21.5%)	0.847
Chronic kidney disease	1575 (16.1%)	1208 (17.4%)	367 (13%)	<0.001
Coronary artery disease	6869 (70.4%)	4723 (68.2%)	2146 (75.9%)	<0.001
Chronic heart failure	2437 (25.0%)	1767 (25.5%)	670 (23.7%)	0.062
Active malignancy	541 (5.5%)	414 (6%)	127 (4.5%)	0.004
Estimated GFR, mL/min/1.73 m^2^	77.2 (58.6, 95.3)	75.8 (57.5, 94.2)	79.9 (62, 97.9)	<0.001
Hemoglobin, mg/dL (*n* = 9361)	13.6 (12.2, 14.8)	13.4 (12, 14.6)	14.1 (12.6, 15.3)	<0.001
Total cholesterol, mg/dl (*n* = 5481)	161 (133, 198)	166 (135, 202)	152 (127, 189)	<0.001
HDL-C, mg/dL (*n* = 5104)	40 (34, 47)	41 (34.2, 48)	39 (33, 45)	<0.001
LDL-C, mg/dL (*n* = 4633)	86 (64, 119)	91 (67, 124)	77 (58, 103)	<0.001
Triglycerides, mg/dL (*n* = 5197)	147 (106, 209)	141 (102, 198)	160 (115, 233)	<0.001
Diabetes duration (year)	14 (7.3, 19.5)	12.5 (5.9, 18.7)	16.8 (11.1, 20.9)	<0.001
Diabetic nephropathy	2509 (25.7%)	1661 (24%)	848 (30%)	<0.001
Diabetic neuropathy	2631 (27.0%)	1678 (24.2%)	953 (33.7%)	<0.001
Diabetic retinopathy	1291 (13.2%)	704 (10.2%)	587 (20.8%)	<0.001
Metformin	6936 (71.1%)	4247 (61.3%)	2689 (95.1%)	<0.001
Insulin	2926 (30%)	1433 (20.7%)	1493 (52.8%)	<0.001
ACS type				
- Unstable angina	2769 (28.4%)	1870 (27%)	899 (31.8%)	<0.001
- NSTEMI/STEMI	6987 (71.6%)	5059 (73%)	1928 (68.2%)	

ACS, acute coronary syndrome; COPD, chronic obstructive pulmonary disease; GFR, glomerular filtration rate.

**Table 2:** Use of glucose-modifying drugs before and after hospitalization with ACS.

		Pre-Hospitalization	Discharge Prescriptions (90 Days)	Post-Hospitalization Prescriptions (12 Months)
Administration				
	
Drug Group				
GLP1-RA				
- Liraglutide		7.1%	6.4% *	8.2% *
- Dulaglutide		6.0%	5.8%	8.6% *
- Semaglutide		0.7%	1.3% *	4.1% *
Overall		13%	13.2%	18.8% *
SGLT2i				
- Empagliflozin		20.2%	29.2% *	36.8% *
- Dapagliflozin		4.1%	6.4% *	8.2% *
Overall		23.9%	33.6% *	42.7% *
GLP1-RA and SGLT2I (both)		7.3%	8.6% *	13.9% *
GLP1-RA and/or SGLT2I (any)		29.6%	38.2% *	47.6% *

* *p* < 0.001 for comparison of time-point to baseline.

**Table 3:** Association of any use of SGLT2Is or GLP1RAs pre- or post-hospitalization with adverse cardiovascular outcomes.

	No. of Events/Patients (%)	Hazard Ratio (95% CI)	*p* Value
**Mortality**			
Crude	2385/9756 (24.4%)	0.457 (0.419–0.500)	<0.0001
Adjusted	2382/9751 (24.4%)	0.724 (0.654–0.801)	<0.0001
**MACE**			
Crude	4648/9756 (47.6%)	1.124 (1.056–1.196)	0.0002
Adjusted	4646/9751 (47.6%)	0.974 (0.909–1.043)	0.4511
**MACE or mortality**			
Crude	5972/9756 (61.2%)	0.956 (0.904–1.012)	0.1194
Adjusted	5968/9751 (61.2%)	0.884 (0.831–0.940)	<0.001

MACE, major adverse cardiovascular events including recurrent hospitalization for ACS, acute revascularization, heart failure, or ischemic stroke.

## Data Availability

The data underlying this article were provided by Clalit Health Services with permission and approval from the institutional ethics committee. Data will be shared upon request to the corresponding author according to permission by Clalit Health Services.

## Supplementary Materials

The following supporting information can be downloaded at https://www.mdpi.com/article/10.3390/jcm13185541/s1, Figure S1: Cumulative incidence of GLP1-RA and/or SGLT2I prescriptions according to the pre-hospitalization use of diabetic drugs. Figure S2: Cumulative 1-year incidence of GLP1-RA and/or SGLT2I prescriptions according to age groups. Table S1: Shift analysis of drug prescriptions from pre-hospitalization users * to any time during first year post-hospitalization.
