# Supplementary material for: Glucose-Lowering Drugs with Proven Cardiovascular Benefit Following Acute Coronary Syndrome in Patients with Type 2 Diabetes: Treatment Gaps and Outcomes
Source: J Clin Med. 2024 Sep 19;13(18):5541. doi: 10.3390/jcm13185541 (PMC11432281; doi:10.3390/jcm13185541)
Supplement: Supplementary file 1 [file jcm-13-05541-s001.zip › jcm-3184348-supplementary.pdf]

Supplemental Data

Supplemental Figure S1: Cumulative incidence of GLP1-RA and/or SGLT2I prescriptions, according to prehospitalization use of the diabetic drugs

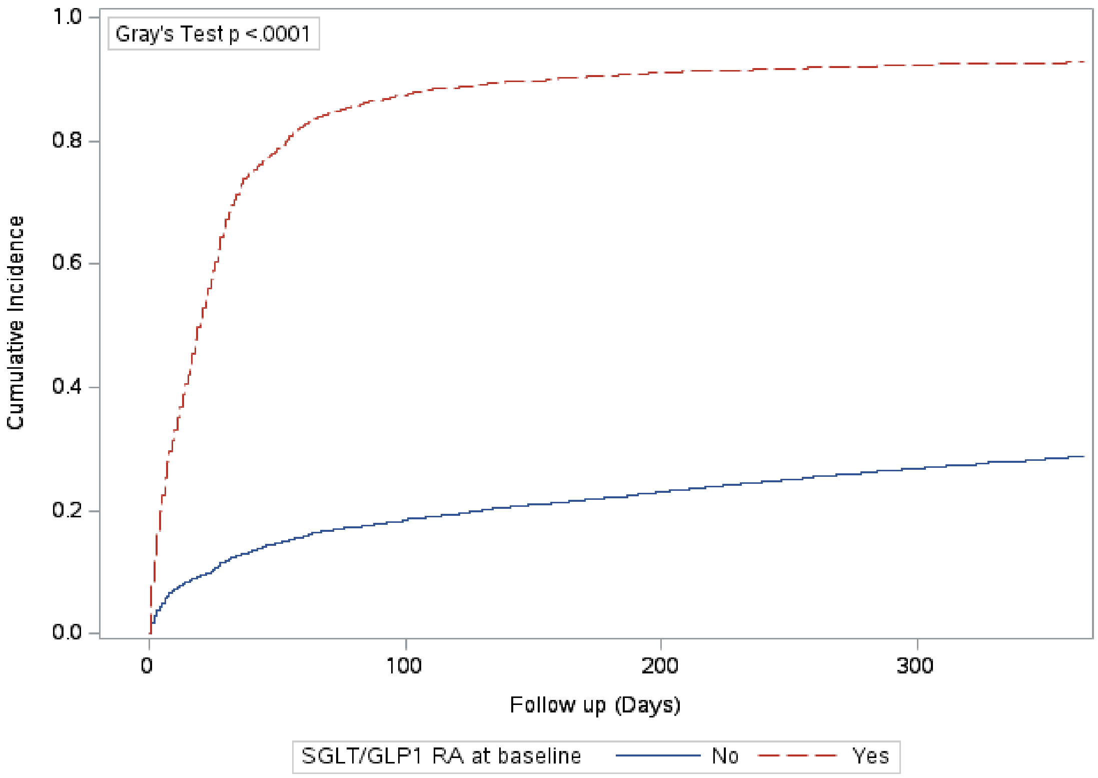

Supplemental Figure S2: Cumulative 1-year incidence of GLP1-RA and/or SGLT2I prescriptions, according to age groups.

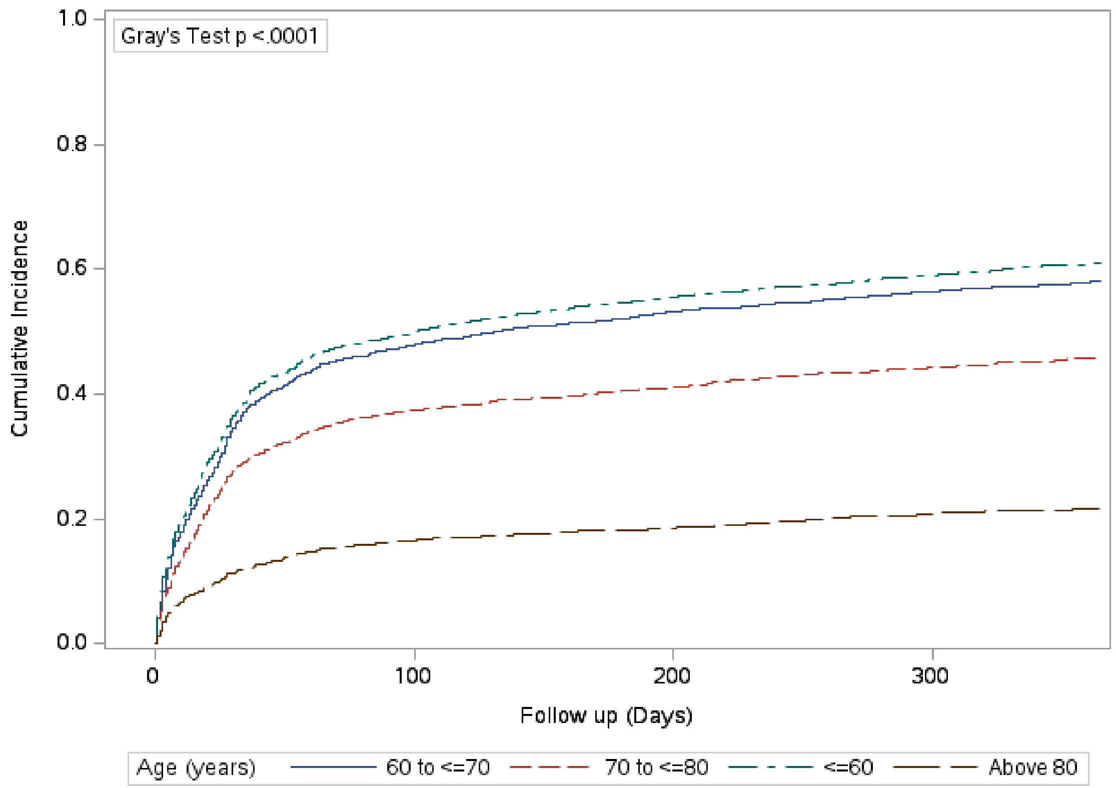

Supplemental Table S1: Shift analysis of drug prescriptions from pre-hospitalization users* to any time during first year post-hospitalization

Shift post-hospitalization	Before hospitalization			
	GLP1RA only	SGLT2I only	GLP1RA & SGLT2I	Total
Added treatment group	197 (37.4%)	263 (17.1%)	0	460 (16.8%)
Removed one treatment group	0	0	99 (14.6%)	99 (3.6%)
No change	247 (46.9%)	1132 (73.8%)	561 (82.7%)	1940 (70.8%)
Stopped treatment	54 (10.2%)	129 (8.4%)	18 (2.7%)	201 (7.3%)
Switched between treatment groups	29 (5.5%)	11 (0.7%)	0	40 (1.5%)
Total	527 100%	1535 100%	678 100%	2740 100%

* At baseline prior to hospitalization, 2,740 patients have used SGLT2I and/or GLP1RA.
